# Group-member selection for RSVP-based collaborative brain-computer interfaces

**DOI:** 10.3389/fnins.2024.1402154

**Published:** 2024-08-21

**Authors:** Yuan Si, Zhenyu Wang, Guiying Xu, Zikai Wang, Tianheng Xu, Ting Zhou, Honglin Hu

**Affiliations:** ^1^Shanghai Advanced Research Institute, Chinese Academy of Sciences, Shanghai, China; ^2^University of Chinese Academy of Sciences, Beijing, China; ^3^Shanghai Frontier Innovation Research Institute, Shanghai, China; ^4^School of Microelectronics, Shanghai University, Shanghai, China

**Keywords:** brain-computer interfaces (BCIs), electroencephalogram (EEG), event-related potentials (ERP), rapid serial visual presentation (RSVP), collaborative brain-computer interfaces (cBCIs), group-member selection

## Abstract

**Objective:**

The brain-computer interface (BCI) systems based on rapid serial visual presentation (RSVP) have been widely utilized for the detection of target and non-target images. Collaborative brain-computer interface (cBCI) effectively fuses electroencephalogram (EEG) data from multiple users to overcome the limitations of low single-user performance in single-trial event-related potential (ERP) detection in RSVP-based BCI systems. In a multi-user cBCI system, a superior group mode may lead to better collaborative performance and lower system cost. However, the key factors that enhance the collaboration capabilities of multiple users and how to further use these factors to optimize group mode remain unclear.

**Approach:**

This study proposed a group-member selection strategy to optimize the group mode and improve the system performance for RSVP-based cBCI. In contrast to the conventional grouping of collaborators at random, the group-member selection strategy enabled pairing each user with a better collaborator and allowed tasks to be done with fewer collaborators. Initially, we introduced the maximum individual capability and maximum collaborative capability (MIMC) to select optimal pairs, improving the system classification performance. The sequential forward floating selection (SFFS) combined with MIMC then selected a sub-group, aiming to reduce the hardware and labor expenses in the cBCI system. Moreover, the hierarchical discriminant component analysis (HDCA) was used as a classifier for within-session conditions, and the Euclidean space data alignment (EA) was used to overcome the problem of inter-trial variability for cross-session analysis.

**Main results:**

In this paper, we verified the effectiveness of the proposed group-member selection strategy on a public RSVP-based cBCI dataset. For the two-user matching task, the proposed MIMC had a significantly higher AUC and TPR and lower FPR than the common random grouping mode and the potential group-member selection method. Moreover, the SFFS with MIMC enabled a trade-off between maintaining performance and reducing the number of system users.

**Significance:**

The results showed that our proposed MIMC effectively optimized the group mode, enhanced the classification performance in the two-user matching task, and could reduce the redundant information by selecting the sub-group in the RSVP-based multi-user cBCI systems.

## 1 Introduction

Brain-computer interfaces (BCIs) are human-machine interaction systems that forge a direct pathway between the user's brain and the external world, bypassing conventional peripheral pathways (Vidal, 1973; Martins et al., 2019; Moioli et al., 2021). Traditional BCIs are designed to provide communication and control solutions for people with severe neuromuscular disorders (McFarland and Wolpaw, 2011). Therefore, the typical BCI applications include brain-controlled spellers (Farwell and Donchin, 1988; Volosyak et al., 2009), brain-controlled wheelchair (Long et al., 2012), and brain-controlled cursor (Li et al., 2008), etc. Recently, some kinds of electroencephalogram (EEG)-based BCIs have been developed for able-bodied users, aiming to enhance human capabilities (Värbu et al., 2022). The Rapid Sequence Visual Presentation (RSVP) based BCI is one of them (Lees et al., 2018).

RSVP focuses on enhancing users' visual search capabilities by utilizing split-second perceptual judgments (Huang et al., 2011; Matran-Fernandez and Poli, 2017). Visual search is a perceptual process that involves scanning the environment to find an item of interest. RSVP-based BCI systems can be employed in designing spellers (Acqualagna and Blankertz, 2013) and for detecting targets, including both static images (Bigdely-Shamlo et al., 2008; Poolman et al., 2008) and videos (Weiden et al., 2012; Rosenthal et al., 2014). The RSVP-based BCIs for target image detection can be applied in counterintelligence and policing for detecting potential threats (Marathe et al., 2015), in medical diagnostics for screening mammograms (Hope et al., 2013), and in geoscientific research for analyzing complex images (Sivarajah et al., 2014). As an example, in counterintelligence and policing work, teams composed of multiple police officers screen large volumes of images daily to identify suspicious individuals or items.

**Figure 1:**
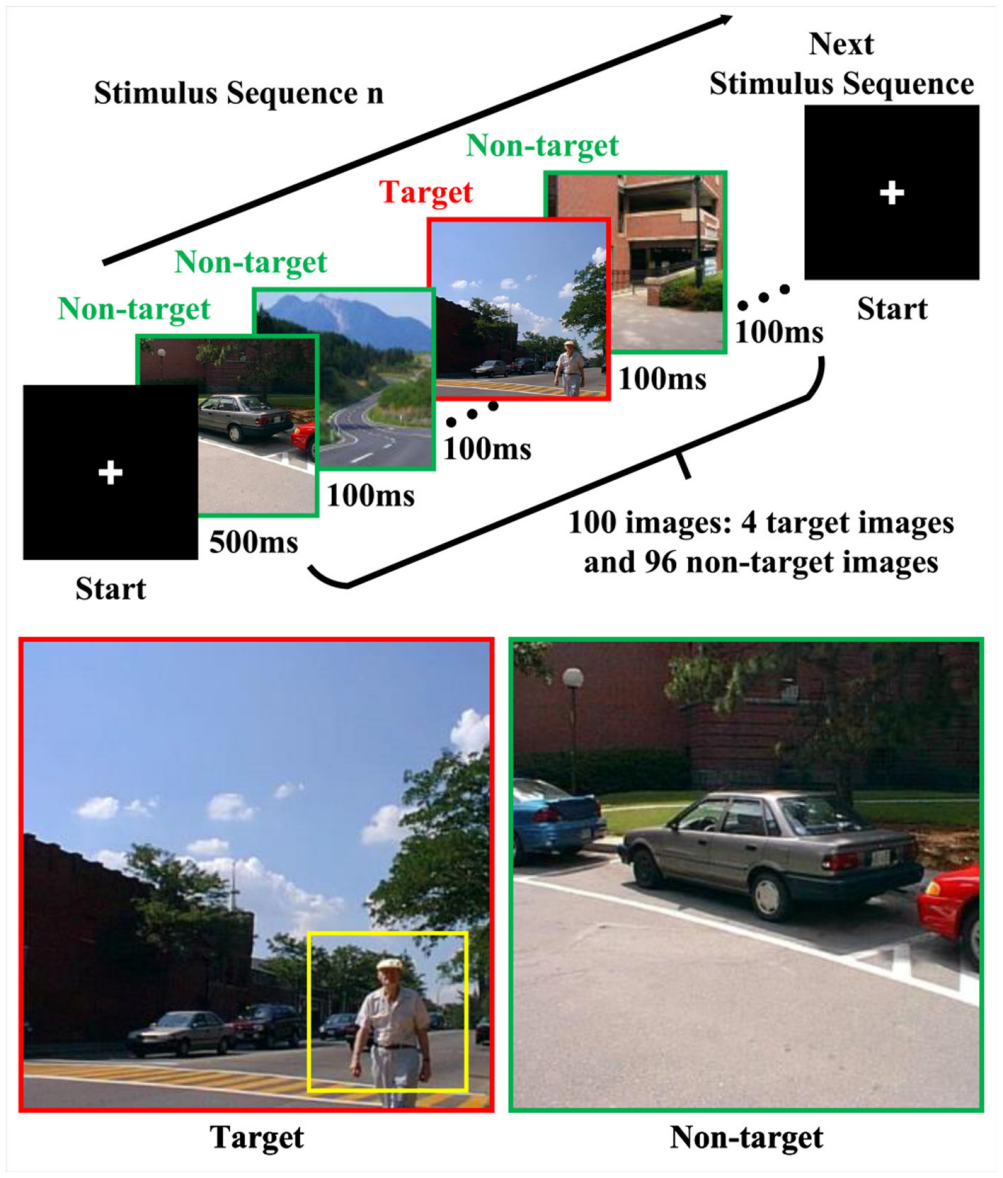
The overview of RSVP stimulation. The target sample is highlighted with a red frame, while non-target samples are marked with a green frame. In a target sample on the left, the human figure is further highlighted with a yellow frame. The images were from Zheng et al. (2020).

As shown in Figure 1, in RSVP-based target image detection, sequences of image stimuli are rapidly presented at a consistent spatial position (Lees et al., 2018). The stream of images comprises frequent non-target samples and infrequent target samples. Compared to non-target samples, target samples are rare, thus a P300 event-related potential (ERP) is elicited when users observe a target sample (Polich and Donchin, 1988). By recording EEG signals and detecting the single-trial ERPs, the RSVP-based BCI systems can distinguish target and non-target images.

Due to the low signal-to-noise ratio (SNR) of EEG signals, the performance of single-trial ERP detection remains limited in RSVP-based BCI systems (Henry, 2006; McFarland and Wolpaw, 2011). Many feature extraction and classification algorithms have been developed to enhance the performance of RSVP-based BCIs (Lees et al., 2018; Lotte et al., 2018; Wu and Wu, 2022; Wang et al., 2023). Typical feature extraction algorithms include xDAWN (Rivet et al., 2009) and SIM (Wu and Gao, 2011). Major classification algorithms include hierarchical discriminant component analysis (HDCA; Sajda et al., 2010), discriminative canonical pattern matching (DCPM; Xiao et al., 2020) and discriminant analysis and classification for interval ERPs (DACIE; Li et al., 2021). With the development of deep learning, the network models such as EEGNet (Lawhern et al., 2018) and its enhanced variants (Zhang et al., 2022) have shown superior classification performance in RSVP-based BCIs.

Furthermore, with the widespread application of BCI technology, the socialization of BCI has emerged as a trend (Hu et al., 2024). In this context, collaborative brain-computer interfaces (cBCIs), which fuse EEG signals from multiple users, have become another approach to enhancing the SNR of EEG signals. In the cBCI paradigm, multiple subjects participate in identical tasks simultaneously (Wang and Jung, 2011; Zheng et al., 2020). The EEG data from these subjects are concurrently recorded and integrated to derive the final classification result. Numerous studies have demonstrated that, when compared to traditional single-brain BCI systems, cBCI systems exhibit superior performance, particularly in terms of speed and accuracy (Wang and Jung, 2011; Stoica et al., 2013; Zhang et al., 2021).

**Figure 2:**
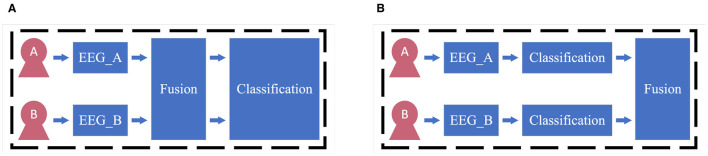
Different types of cBCI. **(A)** The centralized cBCI. **(B)** The distributed cBCI.

In recent years, the exploration and development of multi-user cBCI systems have garnered significant attention in the scientific community. Wang and Jung (2011) categorized cBCI paradigms into centralized cBCI and distributed cBCI as shown in Figure 2, and they proposed three distinct approaches for fusing EEG signals from multiple users. Cecotti and Rivet (2014) further refined the typology of BCI systems, building upon the hybrid BCI (Pfurtscheller et al., 2010) and the cBCI. They suggested a more nuanced categorization of BCIs, based on the diversity of BCI-based tasks and the number of participating subjects. To improve group decisions in cBCIs, Valeriani et al. (2015, 2017a,b) introduced the confidence-weighted voting method specifically designed for cBCI systems. They assessed the confidence level of each group member based on their response times, using this as a measure to evaluate individual capabilities. Subsequently, these confidence levels were used to assign weights to the decisions of each group member, thereby enhancing the overall performance of group decision-making. Furthermore, Salvatore et al. (2022) introduced optimization methods for EEG confidence decoders that take into account both individual capabilities and the overall composition of the group. They also used a hyperparameter to fine-tune the balance between the confidence weights of group members, aiming to strike an optimal balance between accuracy and fairness within the group. However, there is still a gap in research concerning the development of group formation strategies to enhance the performance of cBCI systems.

**Figure 3:**
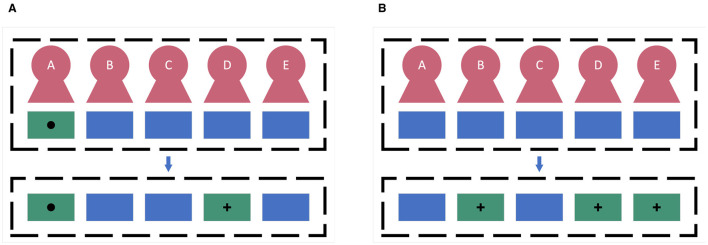
Two kinds of group-member selection tasks. The green boxes with the circle mean a specific user who needs a collaborator. The blue boxes mean users which can be selected and the green boxes with the cross mean the selected collaborator. **(A)** Two-user matching task. **(B)** Sub-group selection task.

Two kinds of group mode optimization tasks are shown in Figure 3. The two-user matching task aims to pair a specific user with the most suitable collaborator to enhance collaborative performance. In conventional two-user cBCI systems, the prevailing approach of randomly matching collaborators is less effective (Matran-Fernandez and Poli, 2014; Zhao et al., 2024). Matran-Fernandez and Poli (2014) proposed a two-user matching method for forming cBCI groups by assessing the similarity of individual performances. They utilized a trained SVM classifier to match users in a two-user cBCI system, focusing on pairing those with minimal dissimilarity in their AUC scores. However, the performance of this method was suboptimal and heavily dependent on the manually set threshold. Upon analyzing various cBCI group modes, Zhao et al. (2024) suggested that the classification AUC might be a criterion for identifying the ideal matched subject in an RSVP-based cBCI system. Yet, their findings indicated that the highest AUC did not always align with the ideal match, leading them to hypothesize that feature distribution similarity might play a role. However, despite this insight, Zhao et al. did not incorporate feature distribution similarity into their selection method, suggesting there may be further opportunities to refine the two-user matching strategy. Moreover, the sub-group selection task aims to select a subset from all collaborators to complete the tasks that are initially assigned to the all-member group. Some studies (Wang and Jung, 2011; Zheng et al., 2020; Zhao et al., 2024) have shown that as the number of subjects increases, there is a significant enhancement in classification accuracy and a substantial reduction in standard deviation. However, with the involvement of more subjects in the system, there is a corresponding increase in hardware and labor costs, as well as computational complexity. Furthermore, the experimental results in Du et al. (2023) showed that the performance of two-user cBCI might outperform that of three-user or four-user cBCI, indicating there might be redundant information between the EEG of the collaborators. Therefore, how to reduce the number of members while still preserving the original critical information is one of the significant challenges in cBCI research.

To address the above drawbacks, this paper proposed a group-member selection strategy for the two-user matching task, which was then further applied to tackle the issue of sub-group selection in RSVP-based cBCI systems. Firstly, in the two-user matching task, we proposed a novel performance score for candidates, taking into account both the individual performance of the candidates and the correlation between a predefined user and the candidates. Drawing inspiration from xDAWN (Rivet et al., 2009, 2011; Xiao et al., 2021), we estimated the signal to signal-plus-noise ratio (SSNR) of each candidate's EEG signal to quantify their individual capabilities. Subsequently, inspired by Liu et al. (2020), we used the Pearson correlation coefficient to estimate the similarity of the ERP waveform from the two users as their collaborative capability. Then the parameter μ was utilized to assign weights to individual capability and collaborative capability, and the user with maximum individual capability and maximum collaborative capability (MIMC) was considered the most suitable collaborator. Secondly, we combined the MIMC strategy with sequential forward floating selection (SFFS) (Pudil et al., 1994) to select the optimal sub-group to reduce the hardware costs, the labor costs, and the computational complexity in multi-user cBCI systems. Thirdly, HDCA (Sajda et al., 2010) was applied for single-trial ERP signal classification. The Euclidean space data alignment (EA) (He and Wu, 2020) was further employed to address the issue of inter-trial variability in cross-session conditions. To the best of our knowledge, it is the first attempt to optimize the group mode by using both the individual capabilities and the collaborative capabilities to improve the system performance in the multi-user RSVP-based cBCI system.

The remainder of this paper is organized as follows. Section 2 introduces the experimental procedure, the dataset, and the proposed methods. Then, the Section 3 presents the classification performance of the proposed method on both the two-user matching task and the sub-group selection task. Finally, the advantages and the limitations of the proposed method and the future research directions are drawn in the Section 4.

## 2 Material and methods

### 2.1 Data description

We used a cross-session RSVP-based cBCI dataset (Zheng et al., 2020) to verify the effectiveness of the proposed method. In this dataset, all 14 subjects were divided into seven fixed groups, each comprising two subjects. These groups respectively participated in two separate sessions of experiments on different days, and each session consisted of three blocks. Each block comprised 14 stimulus sequences, and within each stimulus sequence, 100 street scene images were presented with a presentation rate of 10 Hz in the center of the screen. As shown in Figure 1, these 100 images consisted of four target images which contained humans, and the target images were interspersed within each stimulus sequence, with a minimum time gap of 500 ms between consecutive target images. Thus, there are 1,400 image presentations (56 target and 1,344 non-target image presentations) in one block. Both subjects in a group pressed keys to start a stimulus sequence, and their 62-channel EEG signals were simultaneously recorded. The subjects pressed a button as soon as they detected a target. Regardless of whether the subjects successfully responded, all 56 target trials EEG signals were used for subsequent verification analysis. The experiment was conducted at a sample rate of 1, 000 Hz and a notch filter at 50 Hz was used to remove the power-line noise.

### 2.2 Data preprocessing

The data pre-processing stage includes down-sampling, data segmentation, band-bass filtering, and re-referencing. The EEG data from each subject in the group was down-sampled to 250 Hz. For each block, the down-sampled data was segmented into trials based on event triggers. Each trial included 1, 200 ms of EEG data, starting from 200 ms before the event trigger and extending to 1, 000 ms after the event trigger. Then, the EEG data were band-pass filtered at 2–30 Hz, and the average of all electrodes was used for re-reference. It should be noted that, for each trial EEG signal, we used the 0–500 ms data after the event trigger for group-member selection and classification.

### 2.3 MIMC for two-user matching

In this subsection, we introduced the proposed two-user matching strategy, MIMC. Suppose that *S*_*all*_ = {*s*_1_, *s*_2_, ⋯ , *s*_*N*_*M*__} was the set of all members in a multi-people collaborative group, where *N*_*M*_ was the number of all members. The two-user matching task aimed to pair a specific user, such as *s*_α_∈*S*_*all*_, with the most suitable collaborator. The set of the candidate collaborator was denoted as *S*_*candi*_ = *S*_*all*_−{*s*_α_}, and MIMC was a performance score for the candidate collaborator in *S*_*candi*_.

For a candidate collaborator *s*_β_, $X_{k,\beta }\in {\mathbb{R}}^{N_{C}\times N_{S}}$ was the *k*th trial preprocessed EEG signal, where *N*_*C*_ was the number of channels and *N*_*S*_ was the number of sampling points. The template signal induced by the non-target and target image was respectively denoted by $P_{\beta }^{\left(0\right)}\in {\mathbb{R}}^{N_{C}\times N_{S}}$ and $P_{\beta }^{\left(1\right)}\in {\mathbb{R}}^{N_{C}\times N_{S}}$:


(1) $$\begin{array}{l} P_{\beta }^{\left(0\right)}=\frac{1}{N_{T}^{\left(0\right)}}\overset{N_{T}^{\left(0\right)}}{\underset{i=1}{\sum }}X_{i,\beta }^{\left(0\right)},P_{\beta }^{\left(1\right)}=\frac{1}{N_{T}^{\left(1\right)}}\overset{N_{T}^{\left(1\right)}}{\underset{i=1}{\sum }}X_{i,\beta }^{\left(1\right)}, \end{array}$$

where $N_{T}^{\left(j\right)}$ and $X_{i}^{\left(j\right)}$ respectively represented the number of trials of pattern *j* and the *i*th trial data of pattern *j* ( with *j* = 0, 1).

#### 2.3.1 Individual capability estimation

xDAWN (Rivet et al., 2009, 2011; Xiao et al., 2021) was proposed to maximize the SSNR of P300 evoked EEG signals by estimating spatial filters. For a candidate collaborator *s*_β_, the estimated spatial filter was denoted as


(2) $$U_{\beta }=\underset{U_{\beta }}{\arg\;\max}\rho (U_{\beta }),$$

and the estimated SSNR ρ(*U*_β_) was given by


(3) $$\begin{array}{l} \rho \left(U_{\beta }\right)=\frac{U_{\beta }^{T}{\hat{\Sigma }}_{\beta }^{\left(1\right)}U_{\beta }}{U_{\beta }^{T}{\hat{\Sigma }}_{\beta }U_{\beta }}, \end{array}$$

where


(4) $$\begin{array}{l} {\hat{\Sigma }}_{\beta }^{\left(1\right)}={\left(P_{\beta }^{\left(1\right)}\right)}^{T}P_{\beta }^{\left(1\right)} \end{array}$$

was the covariance matrix of $P_{\beta }^{\left(1\right)}$, and


(5) $$\begin{array}{l} {\hat{\Sigma }}_{\beta }=X_{r,\beta }^{T}X_{r,\beta } \end{array}$$

was the covariance matrix of the reshaped preprocessed EEG signals $X_{r,\beta }\in {\mathbb{R}}^{N_{C}\times \left(N_{T}\times N_{S}\right)}$.

The spatial filter *U*_β_ could be estimated by the generalized eigenvalue decomposition of pair (${\hat{\Sigma }}_{\beta }^{\left(1\right)}$, ${\hat{\Sigma }}_{\beta }$) such that:


(6) $$\begin{array}{l} {\hat{\Sigma }}_{\beta }^{\left(1\right)}U_{1,\beta }=\lambda_{1,\beta }{\hat{\Sigma }}_{\beta }U_{1,\beta }, \end{array}$$

where λ_1, β_ was the largest generalized eigenvalue and *U*_1, β_ was the associated eigenvector. The estimated spatial filter was *U*_β_ = *U*_1, β_, and the estimated SSNR ρ_β_ = λ_1, β_ was the estimated individual capability score for candidate collaborator *s*_β_.

We estimated the ρ of each candidate in *S*_*candi*_ and the individual capability matrix was


(7) $$\begin{array}{l} M_{in,\alpha }=\left[\rho_{1},\rho_{2},\cdots \;,\rho_{N_{candi}}\right], \end{array}$$

where *N*_*candi*_ = *N*_*M*_−1 was the number of candidates for the predefined user *s*_α_.

#### 2.3.2 Collaborative capability estimation

Liu et al. (2020) proposed selecting a subset of source domain subjects to form a new source domain based on the correlation between source and target domain subjects to enhance cross-subject classification performance in RSVP-based BCI systems. Inspired by their work, we utilized the ERP waveform similarity between the predefined user and the candidate as an indicator of their collaborative capability.

Suppose that $X_{k,\alpha ,flat}^{\left(1\right)}\in {\mathbb{R}}^{1\times \left(N_{C}\times N_{S}\right)}$ and $X_{k,\beta ,flat}^{\left(1\right)}\in {\mathbb{R}}^{1\times \left(N_{C}\times N_{S}\right)}$ respectively represented the *k*th trial flattened processed EEG data of the predefined user *s*_α_ and candidate collaborator *s*_β_ induced by the target image. The collaborative capability between the predefined user *s*_α_ and candidate collaborator *s*_β_ was denoted as


(8) $$\begin{array}{l} \sigma_{\alpha \beta }=\frac{1}{N_{T}^{\left(1\right)}}\overset{N_{T}^{\left(1\right)}}{\underset{i=1}{\sum }}corr\left(X_{i,\alpha ,flat}^{\left(1\right)},X_{i,\beta ,flat}^{\left(1\right)}\right), \end{array}$$

where the *corr*(·) was the Pearson correlation. We estimated the collaborative capability between each candidate in *S*_*candi*_ and user *s*_α_, then the collaborative capability matrix was denoted as


(9) $$\begin{array}{l} M_{co,\alpha }=\left[\sigma_{\alpha 1},\sigma_{\alpha 2},\cdots \;,\sigma_{\alpha N_{candi}}\right], \end{array}$$

#### 2.3.3 Two-user matching

Considering the different significance of individual capability and collaborative capability for optimizing group mode, we set the parameter μ to balance the weights assigned to each capability. *Z*-score normalization was conducted to unify the two capabilities, and $M_{in,\alpha }^{*}$ and $M_{co,\alpha }^{*}$ were the unified scores, respectively. The performance score matrix of the candidate for the special user *s*_α_ was


(10) $$\begin{array}{l} M_{\alpha }=\mu M_{in,\alpha }^{*}+\left(1-\mu \right)M_{co,\alpha }^{*}. \end{array}$$

To optimize μ, we employed five-fold cross-validation for each session. Taking session 1 as an example, block 1 of session 1 was used to optimize μ and to execute the two-user matching strategy. There are 1,400 trials of EEG data in block 1, and every 100 trials consists of four target and 96 non-target trials. Given the imbalance of RSVP-based EEG data, we partitioned block 1 into five folds by trial. The first four folds each contained 300 trials, while the last fold contained 200 trials. For each fold, we defined the range of μ as [0, 1], incrementing by a step size of 0.01. Then, for each μ, we used it to construct *M*_*s*_*i*__ for *s*_*i*_∈*S*_*all*_, *i* = 1…*N*_*M*_, and selected the candidate collaborator corresponding to the maximum value in *M*_*s*_*i*__ as the matched collaborator for *s*_*i*_. We applied the average ERP strategy in Section 2.5 and HDCA in Section 2.6 to validate the collaborative capability of these groups selected using this μ. The average AUC of these groups was considered the score for this μ in this fold. We recorded the score for each μ across all folds. The μ with the highest average score in the five folds was considered the optimal result.

### 2.4 SFFS with MIMC for sub-group selection

We implemented the SFFS with MIMC to select the sub-group and reduce the redundant information in the training stage. The pseudo-code was shown in Algorithm 1.

**Algorithm 1:**
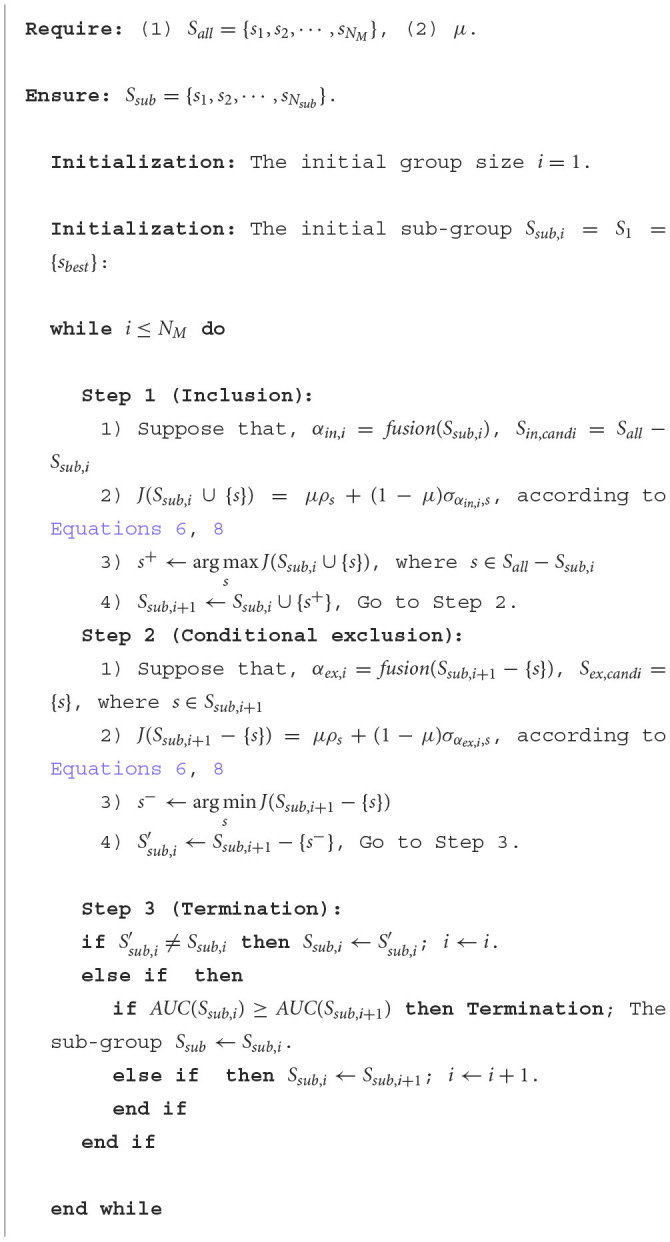
SFFS with MIMC for sub-group selection.

Suppose that *S*_*all*_ = {*s*_1_, *s*_2_, ⋯ , *s*_*N*_*M*__} was the set of all members in a multi-people collaborative group, where *N*_*M*_ was the number of all members. We determined the parameter μ in the two-user matching task, and then the μ was used as the input. The output *S*_*sub*_ = {*s*_1_, *s*_2_, ⋯ , *s*_*N*_*sub*__} was the set of the sub-group selected, where *N*_*sub*_ was the number of members in the selected sub-group.

In the initialization phase, we estimated the individual capabilities of all members according to Equation 6. The user with the best individual capability was used as the initial sub-group *S*_1_ = {*s*_*best*_}. Algorithm 1 selected group members through the following steps until the termination condition was met. In the *i*th iteration, we first treated the inclusion process as a two-user matching problem. The *fusion*(·) referred to the average ERP strategy in Section 2.5. The *S*_*sub, i*_ was considered as a whole, with the members' EEG signals fused using the average ERP strategy. We included a member by maximizing the objective function *J*(*S*_*sub, i*_∪{*s*}). Then, we also treated the conditional exclusion step as a special two-user matching task. When a member *s* was excluded from *S*_*sub, i*+1_, we regarded *S*_*sub, i*+1_−{*s*} as a whole and then *s* and *S*_*sub, i*+1_−{*s*} were viewed as a pair of collaborators. The fused data of *S*_*sub, i*+1_−{*s*} could be treated as a specific member and *s* could be seen as the candidate. The objective function *J*(*S*_*sub, i*+1_−{*s*}) was minimized to remove a member. We used the average ERP strategy in Section 2.5 and HDCA in Section 2.6 to calculate the AUC of *S*_*sub, i*_ and *S*_*sub, i*+1_ in the termination step.

### 2.5 Fusion strategies

In this subsection, we introduced several common fusion strategies for the cBCI paradigm. For the centralized cBCI, the EEG signals or features from multiple users were fused before being input into the classification model. ERP averaging and data combination were two popular centralized fusion strategies. The average ERP strategy averaged the pre-processed EEG signals or extracted features from the collaborators. The combination could be divided into parallel combination and serial combination. Parallel combination concatenated information across the spatial domain, while serial combination concatenated information across the temporal domain. Moreover, the distributed cBCI was based on multiple sub-classifiers and a voting system. In a distributed cBCI system, each user's EEG signals were processed by their respective sub-classifier, which made individual predictions based on those signals. These predictions were then fused using a voting system to form the final decision. Weighted voting was a classic distributed method, in which individual decisions were weighted by the training performance or other confidence weight.

### 2.6 Classification

HDCA (Sajda et al., 2010) was commonly used to classify the ERP component in the RSVP-based BCI system. Suppose that $X^{{\;}^{\prime }}\in {\mathbb{R}}^{N_{T}\times N_{C}^{{\;}^{\prime }}\times N_{S}^{{\;}^{\prime }}}$ was the fused EEG signal and *N*_*T*_, $N_{C}^{{\;}^{\prime }}$, $N_{S}^{{\;}^{\prime }}$ were the number of trials, channels and sample points, respectively. The core idea of HDCA was to train spatial projection matrix $u\in {\mathbb{R}}^{N_{C}^{{\;}^{\prime }}\times W}$ and temporal projection vectors *v*∈ℝ^*W*×1^ for single-trial classification, where *W* was the number of non-overlapping time windows. Suppose that $X_{w}^{{\;}^{\prime }}\in {\mathbb{R}}^{N_{T}\times N_{C}^{{\;}^{\prime }}\times N_{w}}$ was the fused data in the *w*th time window. In this study, we set $N_{w}=\frac{1}{5}N_{S}^{{\;}^{\prime }}$. First, at each time window, Fisher Linear Discriminant (FLD) was used to calculate spatial projection vectors $u_{w}\in {\mathbb{R}}^{N_{C}^{{\;}^{\prime }}\times 1}$,


(11) $$\begin{array}{l} u_{w}=FLD\left(X_{w}^{{\;}^{\prime }}\right),w=1,\cdots \;,W, \end{array}$$

In the *w*th time window, the fused data induced by the non-target and target image was respectively denoted by $X_{w}^{{\left(0\right)}^{\prime }}\in {\mathbb{R}}^{N_{T}^{\left(0\right)}\times N_{C}^{{\;}^{\prime }}\times N_{w}}$ and $X_{w}^{{\left(1\right)}^{\prime }}\in {\mathbb{R}}^{N_{T}^{\left(1\right)}\times N_{C}^{{\;}^{\prime }}\times N_{w}}$. Suppose that $X_{w,r}^{{\;}^{\prime }}\in {\mathbb{R}}^{\left(N_{T}\times N_{w}\right)\times N_{C}^{{\;}^{\prime }}}$ was the reshaped $X_{w}^{{\;}^{\prime }}$, then $X_{w,r}^{{\left(0\right)}^{\prime }}\in {\mathbb{R}}^{\left(N_{T}^{\left(0\right)}\times N_{w}\right)\times N_{C}^{{\;}^{\prime }}}$ and $X_{w,r}^{{\left(1\right)}^{\prime }}\in {\mathbb{R}}^{\left(N_{T}^{\left(1\right)}\times N_{w}\right)\times N_{C}^{{\;}^{\prime }}}$ were the reshaped $X_{w}^{{\left(0\right)}^{\prime }}$ and $X_{w}^{{\left(1\right)}^{\prime }}$ respectively. The template signal of the reshaped fused data in the *w*th time window induced by the non-target and target image was respectively denoted as $P_{w,r}^{\left(0\right)}\in {\mathbb{R}}^{1\times N_{C}^{{\;}^{\prime }}}$ and $P_{w,r}^{\left(1\right)}\in {\mathbb{R}}^{1\times N_{C}^{{\;}^{\prime }}}$:


(12) $$\begin{array}{l} P_{w,r}^{\left(0\right)}=\frac{1}{N_{T}^{\left(0\right)}}\overset{N_{T}^{\left(0\right)}}{\underset{i=1}{\sum }}X_{i,w,r}^{\left(0\right)},P_{w,r}^{\left(1\right)}=\frac{1}{N_{T}^{\left(1\right)}}\overset{N_{T}^{\left(1\right)}}{\underset{i=1}{\sum }}X_{i,w,r}^{\left(1\right)}. \end{array}$$

The total within-class scatter matrix of the fused data in the *w*th time window was


(13) $$\begin{array}{l} S_{W,w,r}=\sum_{i=1}^{N_{T}^{\left(0\right)}}(X_{i,w,r}^{\left(0\right)}-P_{w,r}^{\left(0\right)}){\left(X_{i,w,r}^{\left(0\right)}-P_{w,r}^{\left(0\right)}\right)}^{T}\\ \;+\sum_{i=1}^{N_{T}^{\left(1\right)}}(X_{i,w,r}^{\left(1\right)}-P_{w,r}^{\left(1\right)}){\left(X_{i,w,r}^{\left(1\right)}-P_{w,r}^{\left(1\right)}\right)}^{T}. \end{array}$$

The spatial projection vectors were calculated by


(14) $$\begin{array}{l} u_{w}={\left({\left(S_{W,w,r}\right)}^{-1}{\left(P_{w,r}^{\left(0\right)}-P_{w,r}^{\left(1\right)}\right)}^{T}\right)}^{T}, \end{array}$$

(15) $$\begin{array}{l} u=\left[u_{1},u_{2},\cdots \;,u_{W}\right]. \end{array}$$

For *w*th time window, $Y_{w}\in {\mathbb{R}}^{N_{T}\times 1}$ represents the data after spatial projection:


(16) $$\begin{array}{l} Y_{w}=u_{w}^{T}{\left(X_{w,r}^{{\;}^{\prime }}\right)}^{T}, \end{array}$$

(17) $$\begin{array}{l} Y=\left[Y_{1},Y_{2},\cdots \;,Y_{W}\right], \end{array}$$

where $Y\in {\mathbb{R}}^{N_{T}\times W}$.

Then Fisher Linear Discriminant (FLD) was used to calculate temporal projection vector *v*∈ℝ^*W*×1^ for the signals after spatial projection,


(18) $$\begin{array}{l} v=FLD\left(Y\right). \end{array}$$

Suppose that *P*′(0)∈ℝ^1 × *W*^ and *P*′(1)∈ℝ^1 × *W*^ were the non-target and target templates of the signals after spatial projection.


(19) $$\begin{array}{l} P^{{\;}^{\prime }\left(0\right)}=\frac{1}{N_{T}^{\left(0\right)}}\overset{N_{T}^{\left(0\right)}}{\underset{i=1}{\sum }}Y^{\left(0\right)},P^{{\;}^{\prime }\left(1\right)}=\frac{1}{N_{T}^{\left(1\right)}}\overset{N_{T}^{\left(1\right)}}{\underset{i=1}{\sum }}Y^{\left(1\right)}. \end{array}$$

The total within-class scatter matrix of the signals after spatial projection was


(20) $$\begin{array}{l} S_{W}=\sum_{i=1}^{N_{T}^{\left(0\right)}}(Y^{\left(0\right)}-P^{\prime }(0)){\left(Y^{\left(0\right)}-P^{\prime }(0)\right)}^{T}\\ \;+\sum_{i=1}^{N_{T}^{\left(1\right)}}(Y^{\left(1\right)}-P^{\prime }(1)){\left(Y^{\left(1\right)}-P^{\prime }(1)\right)}^{T}. \end{array}$$

The temporal projection vector was calculated by


(21) $$\begin{array}{l} v={\left({\left(S_{W}\right)}^{-1}{\left(P^{{\;}^{\prime }\left(0\right)}-P^{{\;}^{\prime }\left(1\right)}\right)}^{T}\right)}^{T}, \end{array}$$

Here, *Z*_*th*_ represented the threshold value.


(22) $$\begin{array}{l} Z_{th}=\frac{1}{2}\left(P^{{\;}^{\prime }\left(0\right)}+P^{{\;}^{\prime }\left(1\right)}\right)v \end{array}$$

For a single-trial fused signal $X_{k}^{{\;}^{\prime }}\in {\mathbb{R}}^{N_{C}^{{\;}^{\prime }}\times N_{S}^{{\;}^{\prime }}}$, the data of *w*th window was denoted as $X_{k,w}^{{\;}^{\prime }}\in {\mathbb{R}}^{N_{C}^{{\;}^{\prime }}\times N_{w}}$. When the value of *Z*_*k*_ was greater than *Z*_*th*_, the classification result would equal 1.


(23) $$\begin{array}{l} Z_{k}=\left[u_{1}^{T}X_{k,1}^{{\;}^{\prime }},u_{2}^{T}X_{k,2}^{{\;}^{\prime }},\cdots \;,u_{w}^{T}X_{k,w}^{{\;}^{\prime }}\right]v \end{array}$$

We used HDCA for the within-session classification and the EA-HDCA for the cross-session classification. EA (He and Wu, 2020) was proposed for transfer learning in BCI system. The main idea of EA was to make the data distribution from different domains more similar to improve the transfer performance of the classifier on a new domain. Suppose that the reference matrix *R* was the mean covariance matrix of all *n* trials fused EEG signals of a group:


(24) $$\begin{array}{l} R=\frac{1}{n}\overset{n}{\underset{i=1}{\sum }}X_{i}^{{\;}^{\prime }}X_{i}^{{\;}^{\prime }T} \end{array}$$

To make the data distributions from different sessions more similar, the mean covariance matrices of all sessions should be equal to the identity matrix *I* after alignment. Suppose ${\tilde{X}}_{k}^{{\;}^{\prime }}$ was the *k*th trial after alignment:


(25) $$\begin{array}{l} {\tilde{X}}_{k}^{{\;}^{\prime }}=R^{-\frac{1}{2}}X_{k}^{{\;}^{\prime }} \end{array}$$

(26) $$\begin{array}{l} \frac{1}{n}\sum_{i=1}^{n}{{\tilde{X}}^{\prime }}_{i}{{\tilde{X}}^{\prime }}^{T}{\;}_{i}=\frac{1}{n}\sum_{i=1}^{n}R^{-\frac{1}{2}}{{\tilde{X}}^{\prime }}_{i}{{\tilde{X}}^{\prime }}^{T}{\;}_{i}R^{-\frac{1}{2}}{\tilde{X}}^{\prime }\\ \;=R^{-\frac{1}{2}}\left(\frac{1}{n}\sum_{i=1}^{n}{{\tilde{X}}^{\prime }}_{i}{{\tilde{X}}^{\prime }}^{T}{\;}_{i}\right)R^{-\frac{1}{2}}=I \end{array}$$

For EA-HDCA, the aligned training data were utilized to train the HDCA model, and the aligned test data were used for analysis.

### 2.7 Framework overview

**Figure 4:**
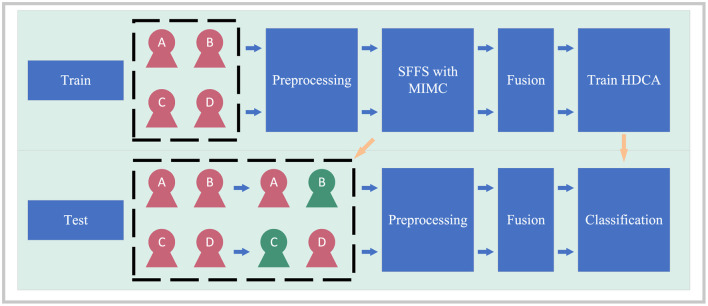
The framework of the cBCI system with group-member selection.

For an example of the sub-group selection task in a four-user centralized cBCI, our proposed framework is shown in Figure 4. For the training stage, the raw EEG signals from all subjects were preprocessed first. Then, the SFFS with MIMC was used to select a sub-group. The EEG data of the selected members were fused in the fusion stage and HDCA was trained to project the single-trial EEG signals to the decision. For the testing stage, the raw EEG signals from the selected members in the training stage were fused and the trained classifier was used to detect the ERP component.

## 3 Results

This section presented the performance of the proposed method in both the two-user matching task and the sub-group selection task. The area under the receiver operating characteristic curve (AUC), true positive rate (TPR), and false positive rate (FPR) were used to analyze the effectiveness of the proposed algorithm. The significant difference was analyzed by one-way ANOVA and paired t-test. The statistical significance was defined as *p*-values < 0.05, and the *Post Hoc* tests were the Least Significant Difference (LSD) corrected in the one-way ANOVA. LSD is a statistical method used in multiple comparisons. It helps to determine which specific group averages are significantly different from one another.

### 3.1 AUC comparison of different fusion strategies

**Figure 5:**
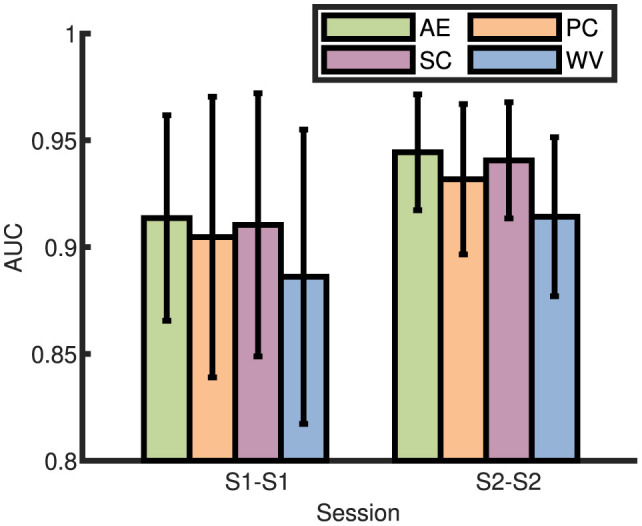
Comparison of the AUC with four different fusion strategies.

In this subsection, we compared the AUC of four different fusion strategies for the seven fixed groups in the dataset to find the best fusion strategy. The AUC of average ERP (AE), parallel combination (PC), serial combination (SC), and AUC-weighted vote (WV) in session 1 (S1) and session 2 (S2) were shown in Figure 5. Taking S1 as an example, block 1 of S1 was used to train the classifier, and block 2 and block 3 of session 1 were used to test (Zheng et al., 2020). For all two sessions, the experiments were denoted as S1–S1 and S2–S2 in within-session conditions, respectively. The one-way ANOVA results showed that there was no significantly better one among the four fusion strategies. Therefore, the average ERP with the highest averaged AUC value was taken as the best fusion strategy for HDCA. For the later analysis, HDCA with average ERP was taken as a model to evaluate the classification performance.

### 3.2 Two-user matching performance

In this subsection, to verify the effectiveness of the proposed method in the two-user matching task, we first compared the MIMC with other conventional group modes in both within-session and cross-session conditions. Then, to analyze the key factors affecting the collaboration capabilities of two users, we compared the MIMC with other potential group-member selection strategies.

#### 3.2.1 Performance comparison between MIMC and conventional group modes

Table 1 showed the AUC, FPR, and TPR of single-user mode, several conventional group modes in cBCI and proposed MIMC in S1–S1 and S2–S2. The fixed grouping was the seven fixed group in the dataset. The random grouping was the group that was randomly selected from all users. The matched group was the group that had the best test performance for each subject (Zhao et al., 2024). One-way ANOVA was conducted for the group modes, which showed significant differences in AUC, TPR, and FPR among these group modes in both S1–S1 and S2–S2 (*p*-values < 0.001). As shown in Table 1, in the within-session conditions, compared with other group modes except for the matched grouping, the MIMC had higher AUC and TPR and lower FPR. Moreover, the one-way ANOVA result showed that there was no significant difference between the MIMC and the matched grouping. It was indicated that the MIMC significantly improved the collaborative performance in two-user matching tasks.

**Table 1:** Within-session performance comparison between MIMC and conventional group mode.

	**S1–S1**			**S2–S2**		
	**AUC (%)**	**TPR (%)**	**FPR (%)**	**AUC (%)**	**TPR (%)**	**FPR (%)**
Single subject	86.80 (6.71)^***^	77.42 (11.47)^***^	3.83 (2.26)^***^	89.86 (5.27)^***^	82.97 (8.78)^***^	3.25 (3.55)^***^
Fixed grouping	91.36 (4.81)^**^	84.82 (8.42)^**^	2.10 (1.22)^*^	94.44 (2.71)^*^	89.92 (5.31)^*^	1.05 (0.18)
Random grouping	92.57 (3.40)^***^	86.94 (6.03)^***^	1.80 (1.05)^**^	95.07 (2.07)^**^	91.33 (3.79)^*^	1.19 (0.92)
Proposed MIMC	**96.97 (0.75)**	**94.71 (1.45)**	**0.78 (0.30)**	**97.01 (0.92)**	**94.64 (1.65)**	**0.62(0.24)**
Matched grouping	96.97 (0.75)	94.71 (1.45)	0.78 (0.30)	97.59 (0.70)	95.73 (1.31)	0.55 (0.21)

The bold fonts indicated the best performance across all group modes except for the matched grouping. The result of the matched grouping was theoretically optimal performance.

Significant difference was analyzed by one-way ANOVA and LSD-adjustment *post hoc* multiple comparisons. The “^*^” indicated the significant difference between the proposed two-user matching strategy and other group modes. “^*^”, “^**^”, and “^***^” meant *p*-values < 0.05, *p*-values < 0.01, and *p*-values < 0.001, respectively. Without “^*^” meant *p*-values >0.05.

**Figure 6:**
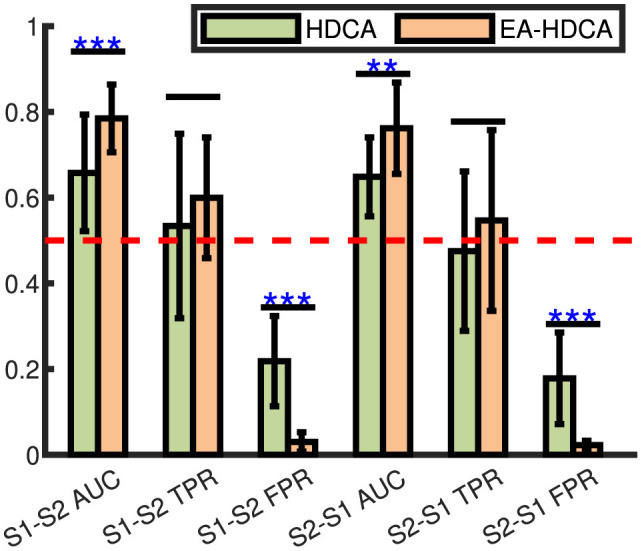
Comparison of the performance with HDCA and EA-HDCA in cross-session situations. The “^*^” indicated the significant difference between HDCA and EA-HDCA. “^**^” and “^***^” meant *p*-values < 0.01 and *p*-values < 0.001, respectively. Without “^*^” meant *p*-values >0.05.

The group-member selection process in the cBCI system was labor-intensive and time-consuming for the whole group due to the need for substantial computational resources to collect and analyze EEG signals from all potential collaborators. Cross-session analysis was necessary to validate the robustness of the selected groups, ensuring that a group selected on one day could be effectively used on other days. This avoids the need for repeated labor-intensive and time-consuming selection processes before each session. The cross-session condition was denoted as S1–S2 and S2–S1, in which block 1 of one session was used to train, and block 2 and block 3 of another session were used to test. To ensure that the experimental results are not influenced by the classifier's own cross-session capabilities, we conducted cross-session experiments and used EA-HDCA as the classifier. This was crucial because a stable and reliable classifier allows us to accurately assess the robustness of the selected collaborative groups across different sessions, eliminating variability introduced by the classifier itself and ensuring that any observed performance differences are due to the collaborative groups rather than the classifier's instability. Figure 6 compared the cross-session classification performance of the HDCA and EA-HDCA under the single subject mode. In Figure 6, the red dashed line represented the chance level, and the TPR of HDCA was lower than the chance level in S2–S1. It was suggested that the HDCA was not effective in overcoming the ERP variability among different sessions. Moreover, the paired *t*-test results showed that the EA-HDCA had higher AUC values and lower FPR values than HDCA. Therefore, for the later analysis of cross-session conditions, EA-HDCA with ERP averaging was used to evaluate the classification performance.

As shown in Table 2, similar to the within-session experiment results, the one-way ANOVA results showed that there were significant differences in AUC, TPR, and FPR among these group modes in both S1–S2 and S2–S1 (*p*-values < 0.001) and the proposed MIMC had better performance compared with other group modes. There was no significant difference between the MIMC and the matched grouping in cross-session conditions. It was indicated that the member combinations selected using MIMC on one day could be used on the other days. It could contribute to time reduction and process simplification of the cBCI system calibration. This might be attributed to the fact that, although different subjects exhibit varied neural responses to the same stimulus, these individual differences may demonstrate temporal consistency. For example, the group that had superior performance in one session was likely to exhibit superior performance in another session.

**Table 2:** Cross-session performance comparison between MIMC and conventional group mode.

	**S1–S2**			**S2–S1**		
	**AUC (%)**	**TPR (%)**	**FPR (%)**	**AUC (%)**	**TPR (%)**	**FPR (%)**
Single subject	78.47 (7.88)^***^	59.95 (14.08)^***^	3.02 (2.26)^***^	76.19 (10.66)^**^	54.66 (21.07)^**^	2.27 (0.99)^***^
Fixed grouping	80.47 (7.54)^**^	62.63 (14.13)^**^	1.68 (1.29)^*^	77.39 (8.94)^*^	55.87 (17.64)^*^	1.08 (0.34)
Random grouping	82.35 (6.28)^***^	65.89 (12.28)^***^	1.19 (1.03)^*^	80.16 (7.70)^*^	61.32 (15.32)^*^	1.00 (0.47)^*^
Proposed MIMC	**89.28 (4.71)**	**78.89 (9.41)**	**0.33 (0.14)**	**85.28 (7.61)**	**71.17 (15.07)**	**0.62 (0.26)**
Matched grouping	90.55(3.87)	81.70(7.63)	0.60(0.30)	90.60(2.51)	81.82(5.05)	0.63(0.46)

The bold fonts indicated the best performance across all group modes except for the matched grouping. The result of the matched grouping was theoretically optimal performance.

Significant difference was analyzed by one-way ANOVA and LSD-adjustment *post hoc* multiple comparisons. The “^*^” indicated the significant difference between the proposed two-user matching strategy and other group modes. “^*^”, “^**^”, and “^***^” meant *p*-values < 0.05, *p*-values < 0.01, and *p*-values < 0.001, respectively. Without “^*^” meant *p*-values >0.05.

#### 3.2.2 Performance comparison between MIMC and other group-member selection methods

We also compared the proposed MIMC with other group-member selection methods. The Individual Performance Dissimilarity (IPD) (Matran-Fernandez and Poli, 2014) method used the AUC dissimilarity between two users as the performance score to pair users. Drawing inspiration from Zhao et al. (2024), we implemented the Best Individual Performance (BIP) approach, wherein the user exhibiting the highest AUC value was identified as the optimal collaborator. We also considered that the group with the best collaborative AUC on the training stage would constitute an effective group mode, and implemented the Best Collaborative Performance (BCP) method. Note that, we used the HDCA with average ERP to implement these AUC-based methods.

Table 3 showed the comparison results. The one-way ANOVA results illustrated that there were significant differences among the four group-member selection methods in S1–S1 (*p*-values < 0.001) and S2–S2 (*p*-values < 0.05). In S1–S1, according to the *Post Hoc* test with LSD adjustment results, the proposed MIMC provided higher AUC and TPR than IPD and BCP and lower FPR than IPD. MIMC and BIP selected the same collaborators for each user. The IPD provided the worst classification performance among the four methods (*p*-values < 0.05). In S2–S2, the MIMC provided higher AUC and TPR than the other three methods and provided lower FPR than IPD and BCP. There were no significant differences between IPD, BIP, and BCP in S2–S2. Therefore, the proposed MIMC selected better collaborators for each subject and improved the classification performances.

**Table 3:** Performance comparison between MIMC and other group-member selection methods.

**Session**	**S1–S1**			**S2–S2**		
	**AUC (%)**	**TPR (%)**	**FPR (%)**	**AUC (%)**	**TPR (%)**	**FPR (%)**
IPD	91.75 (4.03)^***^	85.46 (7.10)^***^	1.95 (1.10)^***^	94.88 (2.63)^**^	90.88 (4.90)^**^	1.13 (0.53)^**^
BIP	**96.97 (0.75)**	**94.71 (1.45)**	**0.78 (0.30)**	95.35 (1.41)^*^	91.58 (2.71)^*^	0.88 (0.41)
BCP	95.03 (2.20)^*^	91.26 (3.88)^*^	1.20 (0.89)	95.20 (1.90)^*^	91.45 (3.57)^*^	1.06 (0.56)^*^
proposed MIMC	**96.97 (0.75)**	**94.71 (1.45)**	**0.78 (0.30)**	**97.01 (0.92)**	**94.64 (1.65)**	**0.62 (0.24)**

The bold fonts indicated the best performance across all group-member selection methods.

Significant difference was analyzed by one-way ANOVA and LSD-adjustment *post hoc* multiple comparisons. The “^*^” indicated the significant difference between the proposed two-user matching strategy and other group selection methods. “^*^”, “^**^”, and “^***^” meant *p*-values < 0.05, *p*-values < 0.01, and *p*-values < 0.001, respectively. Without “^*^” meant *p*-values >0.05.

Additionally, we compared IPD, BIP, and BCP with other group modes. The one-way ANOVA with LSD adjustment results showed that there was no significant difference between IPD and random grouping. It might be because although the two subjects had similar AUC, it did not necessarily mean they had more similar neural responses. BIP and BCP also did not demonstrate a significant difference when compared to random grouping in session 2. One possible reason could be the AUC-based methods suffered from over-fitting. For BIP, as shown in Table 1, the decrease in individual performance variability among subjects in session 2 could be another reason.

#### 3.2.3 Performance comparison between different values of μ

In MIMC, the parameter μ played a crucial role in balancing the contribution of individual capability and collaborative capability. As shown in Table 4, the one-way ANOVA results indicated there were significant differences in AUC among different values of μ in S1–S1 (*p*-values < 0.001), S1–S2 (*p*-values < 0.05), and S2–S1 (*p*-values < 0.05). The *Post Hoc* tests with LSD revealed that, in S1–S1 and S1–S2, the AUC for the method focusing solely on collaborative capabilities (μ = 0) was surpassed by both other approaches. Similarly, in S2–S1, the approach centered exclusively on individual capabilities (μ = 1) demonstrated lower AUC values compared to its counterparts. The sub-optimal performance of (μ = 0) and (μ = 1) could likely be attributed to the participants' varying familiarity with the RSVP paradigm across sessions. In session 1, subjects' unfamiliarity with the RSVP paradigm led to considerable differences in their individual capabilities. Under these conditions, methods emphasizing individual capabilities could potentially achieve higher AUC values. However, in session 2, as participants became more familiar with the RSVP paradigm, the emphasis shifted toward the importance of collaborative capability. The optimal μ value adeptly combined both of them, enhancing system performance. Our experimental results determined optimal μ values to be 0.86 for session 1 and 0.64 for session 2, suggesting that individual capability played a more important role than collaborative capability.

**Table 4:** AUC (%) of different values of μ.

	**S1–S1**	**S2–S2**	**S1–S2**	**S2–S1**
μ = 0	93.30 (3.57)^***^	96.72 (1.01)	84.51 (6.44)^*^	**85.70 (6.80)**
Optimized μ	**96.97 (0.75)**	97.01 (0.92)	**89.28 (4.71)**	85.28 (7.61)
μ = 1	**96.97 (0.75)**	**97.25 (0.77)**	**89.28 (4.71)**	78.32 (7.14)^*^

The bold fonts indicated the best performance across three different values of μ.

Significant difference was analyzed by one-way ANOVA and LSD-adjustment *post hoc* multiple comparisons. The “^*^” indicated the significant difference between the optimized μ and other values of μ. “^*^” and “^***^”meant *p*-values < 0.05 and *p*-values < 0.001, respectively. Without “^*^” meant *p*-values >0.05.

### 3.3 Sub-group selection performance

In this subsection, we extended the proposed MIMC from the two-user cBCI system to the multi-user cBCI system. The all-member mode was the group with all 14 subjects in the dataset. The sub-group mode was the sub-group selected by the proposed SFFS with MIMC from the 14 subjects. The AUC, TPR, FPR, and the group size of the all-member mode and sub-group mode in both within-session conditions and cross-session conditions were shown in Table 5. In general, the all-member mode provided slightly higher AUC and TPR with lower FPR compared to the sub-group mode. However, the group size of the two modes indicated that the group member number of the sub-group drastically reduced. Specifically, the selected sub-group consisted of merely one-seventh of the total subjects in session 1 and two-seventh in session 2, respectively. Therefore, the proposed method could sacrifice a slight amount of system performance to substantially reduce the number of members in a multi-user cBCI system. Notably, in the S2–S1, the sub-group has a higher AUC and TPR than the all-member mode, indicating that if a better sub-group was selected, the performance might be improved in the multi-user cBCI system.

**Table 5:** Classification performance with all-member and sub-group selected by SFFS with MIMC.

	**All-member**				**SFFS with MIMC**			
	**AUC (%)**	**TPR (%)**	**FPR (%)**	**Num**	**AUC (%)**	**TPR (%)**	**FPR (%)**	**Num**
S1–S1	98.53	97.32	0.26	14	97.97	96.43	0.48	2
S2–S2	99.53	99.11	0.04	14	99.05	98.21	0.11	4
S1–S2	98.21	96.43	0.00	14	95.93	91.96	0.11	2
S2–S1	94.14	88.39	0.11	14	95.01	90.18	0.15	4

**Figure 7:**
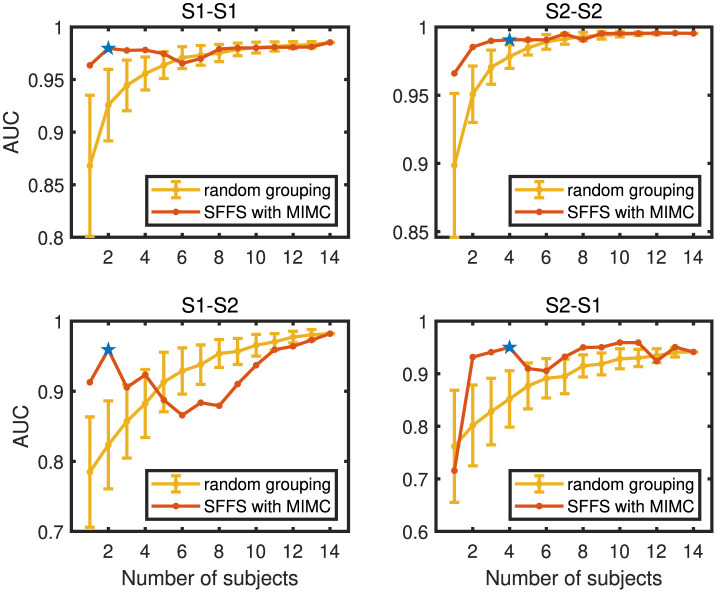
Performance of the random grouping and SFFS with MIMC in the sub-group selection task. The blue stars represented the group size and AUC of the selected sub-group using SFFS with MIMC.

As shown in Figure 7, we discussed the effects of the number of group members on AUC under the random grouping mode and the MIMC mode in the multi-user cBCI system. For random grouping mode in multi-user cBCI, with a group size of *n*, we formed a group by randomly choosing *n* participants from all 14 subjects. Considering the computational complexity, for a group size of n, the maximum number of random combinations generated from choosing *n* out of 14 was capped at 100 to manage the variety of possible combinations. As the number of collaborators within the group increased, the AUC demonstrated an upward trend under the random grouping mode. For the group-member selection mode, we selected a sub-group from all 14 subjects using SFFS with MIMC. To analyze the effectiveness of the proposed method, Figure 7 showed the AUC for the selected sub-group terminating at the predefined number of subjects first. Under this termination policy, SFFS with MIMC significantly outperformed random grouping with a smaller predefined number, such as 2–4 members. This trend highlighted the MIMC strategy's superior initial selection of team members, where the benefits of the selection strategy were maximized with a limited number of collaborators. This aligned with our original intention of striking a balance between the size of the group and the collaborative performance. The blue stars represented the AUC and group size of the selected sub-group using SFFS with MIMC, indicating that SFFS with MIMC effectively balanced the number of selected members and the collaborative AUC.

## 4 Discussion

In this section, we discussed and concluded the effectiveness and the limitations of the proposed method, and introduced the possible future research directions.

### 4.1 Effectiveness of proposed method

In this study, we proposed the MIMC strategy to pair collaborative groups for the two-user matching task. To verify the effectiveness of the MIMC strategy in the two-user matching task, we first compared MIMC with other conventional group modes under both within-session and cross-session conditions. In both conditions, MIMC achieved higher AUC and TPR, lower FPR compared to other group modes, and showed no significant difference from the matched grouping. These results demonstrated that the MIMC strategy significantly improves collaborative performance. Secondly, we compared MIMC with other existing and potential group-member selection strategies, including IPD (Matran-Fernandez and Poli, 2014), BIP (Zhao et al., 2024), and BCP. The IPD strategy (Matran-Fernandez and Poli, 2014) selected the candidate whose AUC is most similar to the specific user. The BIP strategy (Zhao et al., 2024) selected the candidate with the highest AUC. In contrast, we estimated the SSNR of each candidate's EEG signal to quantify their individual capabilities and used the Pearson correlation coefficient to estimate the similarity of the ERP waveform from the two users as their collaborative capability. The proposed MIMC strategy selected the collaborator with the maximum individual capability and maximum collaborative capability. The comparison results showed that the MIMC outperformed other group-member selection strategies. Thirdly, we compared the classification performance with different values of μ. The results showed that the optimized μ could balance the contribution of individual capability and collaborative capability. Furthermore, we combined the MIMC strategy with SFFS to address the issue of sub-group selection in RSVP-based cBCI systems. The results showed that the proposed method could sacrifice a slight amount of system performance to substantially reduce the number of members in a multi-user cBCI system.

### 4.2 Limitations and future research

Although the proposed method can effectively optimize the group mode in the RSVP-based cBCI system, there are three major limitations to this method. First, this study is based on public datasets and requires further validation through online experimentation. Another limitation is that only a group with fourteen collaborators was included in our experiment. In follow-up studies, the EEG data of more groups and the EEG data of groups with more members should be recorded, and the proposed method should be tested on these groups. Finally, it should be noted that the pseudo-multi-user collaboration experiments might be not identical to the actual multi-user collaboration experiments. It is necessary to verify our method in a real-world multi-user cBCI system.

Additionally, the development of cBCI technology is expected to support the innovation of the next generation of human-computer interaction systems (Jiang et al., 2019; Gu et al., 2021). We believe that the Internet of Brains (IoB) is an essential development direction for multi-user human-computer interaction systems (Martins et al., 2019; Moioli et al., 2021; Hu et al., 2024). The IoB can be considered as the next step in the Internet of Things (IoT) (Silva et al., 2017). In the IoB, multiple brains are seamlessly connected to the wireless network as nodes of the communication grid. Compared with the IoT, the IoB with “brain in the loop” would allow more direct interactions between users and networks. The future cBCI-based IoB should be expanded in the following aspects: (1) Group-member selection strategies: The random grouping mode results in poor collaborative performance. Therefore, cBCI systems should optimize the group mode based on both individual capabilities and relationships between the users to improve the group's performance. (2) Task allocation strategies for single BCI paradigm (Gu et al., 2021): The users performed the same task together in the existing cBCI system, which did not fully consider the rationality of task allocation. The division-of-work strategies that reduced the number of users recognizing the same instructions could reduce individual workload and improve overall system performance. (3) Task allocation strategies for hybrid BCI (Cecotti and Rivet, 2014): Cecotti and Rivet proposed the cooperative-hybrid BCI, which involved multi-user and multi-paradigm. We assume that the collaborative has varying adaptability to different BCI paradigms. Based on this hypothesis, task allocation for hybrid BCI should consider user adaptability to different paradigms, such as the P300 speller and RSVP, to further optimize the division-of-work strategies.

## 5 Conclusion

In this work, we introduced a novel group-member selection strategy that considered both individual capability and collaborative capability within the RSVP-based cBCI system. The effectiveness of the proposed MIMC was demonstrated through its application to both the two-user matching task and the sub-group selection task. For the two-user matching task, the classification results showed that the specially optimized group mode surpasses traditional random grouping and other group-member selection methods based on AUC. Furthermore, for the sub-group selection task, the implementation of SFFS with MIMC successfully achieved the trade-off between maintaining performance and enhancing system efficiency. Consequently, our research contributes to the practical advancement of RSVP-based cBCI systems for real-world applications.

## Data availability statement

The original contributions presented in the study are included in the article/supplementary material, further inquiries can be directed to the corresponding author.

## Author contributions

YS: Data curation, Formal analysis, Methodology, Project administration, Writing – original draft. ZhW: Conceptualization, Formal analysis, Methodology, Writing – review & editing. GX: Validation, Writing – review & editing, Formal analysis. ZiW: Validation, Writing – review & editing, Formal analysis. TX: Writing – review & editing, Validation. TZ: Validation, Writing – review & editing. HH: Conceptualization, Supervision, Writing – review & editing.
